# Exploratory Examination of How Race and Criminal Record Relate to Housing Instability Among Domestic Violence Survivors

**DOI:** 10.1177/08862605211042626

**Published:** 2021-09-04

**Authors:** Jasmine Engleton, Cris M. Sullivan, Noora Hamdan

**Affiliations:** 1 Michigan State University, East Lansing, MI, USA

**Keywords:** housing instability, intimate partner violence, criminality, race

## Abstract

Housing instability is a critical concern in the United States, and domestic violence (DV) survivors are a group at high risk for experiencing housing instability or of becoming unhoused. Prior research has also identified having a criminal record (CR) as being a major barrier to obtaining stable housing, and this is truer for Black and Latinx people compared to their White counterparts. No study has examined whether comparable trends exist among survivors of DV, a group also at elevated risk of having a CR, sometimes related to their experience of abuse. The current exploratory study included 305 unhoused or unstably housed female DV survivors who had sought out DV support services. Multivariate regressions explored if survivor race and CR were separately linked to greater housing instability. CR was then explored as a potential moderator in the relation between race and housing instability. Results revealed that DV survivors with a CR faced greater housing instability than those without a CR, Black and Latina survivors experienced greater housing stability than did White survivors, and CR did not moderate the relation between race and housing instability. The racial differences were unexpected and are discussed in light of methodological limitations. This is the first study to date to explore the role of CR possession on housing instability for DV survivors.

## Introduction

Housing instability is a pervasive issue affecting millions of people in the United States (Joint Center for Housing Studies of Harvard University, 2019). The inability to obtain or maintain safe and stable housing can manifest as being unable to afford rent, spending more than 50% of one’s income on housing, having to move frequently, living in substandard or dangerous housing, experiencing overcrowding, or being at risk of eviction and homelessness (Adams et al., 2018; Gilroy et al., 2016). Domestic violence (DV) survivors are especially vulnerable to experiencing housing instability (Levin et al., 2004; Pavao et al., 2007). DV is a pattern of abuse that consists of emotional, physical, or sexual abuse, controlling behaviors, or economic actions by one partner to maintain power and control over the other (Breiding et al., 2015). Though DV is a leading cause of family homelessness (Pavao et al., 2007), housing instability may not be experienced by all DV survivors equally. Previous studies looking at the general population have consistently shown that disparities in housing instability exist by race and possession of a criminal record (CR; Desmond, 2016; Evans & Porter, 2014; Phinney et al., 2007). People of Color (POC) who have a CR show disparate experiences of housing instability compared to their White counterparts (Lucius, 2018; Olivet et al., 2018) and these patterns may be similar among DV survivors.

## Race and Housing Instability

Previous studies have shown that POC are overrepresented in unstably housed populations in the U.S. (Desmond, 2016; Evans et al., 2019; Olivet et al., 2018). There are a myriad of structural and systemic factors that make it more difficult for POC to secure safe and stable housing, including but not limited to housing and employment discrimination, barriers to economic mobility, income inequality, and hypercriminalization (Olivet et al., 2018, 2021). POC experience higher rates of housing cost burden (i.e., spending more than 30% of income on housing), eviction, and homelessness compared to White people of similar incomes (Desmond, 2012; Joint Center for Housing Studies of Harvard University, 2019; Olivet et al., 2018). Additionally, POC incur more lease violations and fines that can also contribute to eviction and homelessness (Olivet et al., 2018). Compared to White groups, Latinx and African Americans face eviction almost twice as often (Desmond, 2012). Black women are more than twice as likely to be evicted as Black men and three times as likely to be evicted as White women despite comprising a smaller tenant percentage (Desmond, 2012).

### Race, Criminal Record, and Housing Instability

POC and people with CRs experience greater instances of housing instability than do White people and those without CRs (Desmond, 2012; Evans & Porter, 2014; Evans et al., 2019; Phinney et al., 2007). POC who hold CRs are at greater housing instability risk than are either POC without CRs or White people with CRs (Couloute & Kopf, 2018). Similar to, yet unique from racially focused oppression, people with CRs face structural barriers like social stigma and discriminatory housing policies, which contribute to housing instability (Olivet et al., 2021).

### Domestic Violence Victimization and Criminal Background

While scant research has been conducted examining the relationship between DV victimization and criminal behavior, limited prior studies have shown a link between the two (Beijer et al., 2018; Iratzoqui & Cohn, 2020). Some survivors are coerced into criminal activity by the abusive partner, and others may engage in illegal behavior as a coping mechanism (Beijer et al., 2018). This might involve stealing due to a lack of control over finances or use of illegal drugs to quell mental health struggles related to being abused. Still others participate in criminality for reasons unrelated to DV (Herrera et al., 2011). Regardless, DV survivors who have such a record face another housing barrier and are more likely to be homeless (Beijer et al., 2018; Iratzoqui & Cohn, 2020).

Housing instability may differ among DV survivors of color and/or those who have a CR due to forces housed within historical, political, material, and social contexts (Crenshaw, 1990). Studies have independently shown that POC face greater housing instability than do their White counterparts, that having a CR is a housing barrier, and that housing instability is intimately tied to DV (Desmond, 2012; Evans & Porter, 2014; Evans et al., 2019; Olivet et al., 2018, 2021; Phinney et al., 2007). Given then, it is important to explicitly consider the interrelations among these factors for DV survivors. The current exploratory study assessed the extent to which severity of housing instability was associated with race/ethnicity and/or having a CR, among a sample of unstably housed, female DV survivors seeking DV support services. We hypothesized that (a) Black and Latina survivors would report greater housing instability than would White survivors; (b) survivors with a CR would report greater housing instability than those without a CR; and (c) having a CR would moderate the relationship between race and housing instability.

## Method

Data for the present study were derived from the baseline time point of a longitudinal study evaluating a housing intervention for DV survivors, when survivors first sought DV support services. Survivors were recruited from five DV agencies in the Pacific Northwest. Study eligibility included being at least 18 years of age, having recently experienced DV, and self-identifying as experiencing housing instability or being unhoused. Data were collected through face-to-face, structured interviews, and participants received $50 for their time.

### Measures

*Race/ethnicity.* Participants were asked “What is your race/ethnicity?” and could choose as many race/ethnicity options as they felt appropriate. In total, 47% of survivors identified as White (*n* = 142), 36% as Latina (*n* = 110) and 17% as Black (*n* = 53). The remaining 23% of the sample identified as multiracial or another race and were excluded because their sample sizes were too small to create meaningful groups.

*Domestic violence victimization.* DV was measured by the Composite Abuse Scale (Loxton et al., 2013). The scale consists of four subscales, with a total of 31 items that capture survivors’ experiences of stalking/harassment, sexual, physical, and emotional abuse. Response options were on a Likert scale, ranging from (0) never to (5) daily (α = 0.95).

*Criminal record.* CR background was measured with one dichotomous (yes/no) item: “Do you have a criminal charge that would show up on a background check?”

*Housing instability*. A 7-item Housing Instability Scale was adapted from the 10-item Housing Instability Index (Rollins et al., 2012). The adapted measure included six items from the original index, excluding those about landlord issues since this question was not relevant to our sample. Example items include the following: “In the past six months, have you had difficulty (or were you unable to) pay for your housing?” and “In the past six months, have you had to live somewhere you did not want to live?” An additional item was added to the total 7-item measure: “Have you been homeless or had to live with family or friends to avoid being homeless in the past six months?” For each item, 0 *= more stable* and 1 = *less stable*. Total scores ranged from 0 to 7, with higher scores indicating greater housing instability (Cronbach’s alpha = 0.71).

### Analyses

Multiple regression analyses were conducted controlling for survivor age and DV victimization severity, as these have been shown in prior studies to both correlate with and impact housing stability (Pavao et al., 2007). A power analysis (α = .05; 1 – β = .80) revealed that the sample size was sufficient for detecting a small effect size (*d* = .08; Faul et al., 2009). In total, two dummy variables for DV survivor race were computed and White DV survivors were used as the reference group in this modeling (i.e., Latina dummy variable; Black dummy variable). This step was completed to test whether Black and Latina survivors differed from White survivors in how race was related to their level of housing instability. A stepwise approach was then used where all control variables were entered into the first step of the regression and the independent variable (survivor race), proposed moderator (survivor CR) and the interaction term between race and CR (Race × CR) were entered into the second step.

## Results

Survivors were from low income families with 79% making less than 35,000 year. In total, 29% of the sample reported having a CR 38% of the White survivors (*n* = 54), 16% of the Latina survivors (*n* = 18), and 28% of the Black survivors (*n* = 15). Ages ranged from 19 to 62 years old (*M* = 34.6; *SD* = 8.99) with Latina survivors being approximately two and a half years younger than Black and White survivors. In all regression models, DV was a predictor of housing instability, while age was not.

Race was associated with housing instability for all groups but in the opposite manner than predicted. Both Latina (*B* = –.515; *p =* .009) and Black (*B* = –.741, *p* = .005) DV survivors experienced less housing instability than did White DV survivors (*B* = .580, *p* = .002). CR was a significant predictor of housing instability among Black and White DV survivors (*B* = .577, *p* = .007; *B* = .719, *p* = .009) but not for Latina survivors. These findings can be seen in Table 1.

**Table 1.** Regression Models.

	*B*	Std. Error	β	*t*	Sig.
Model					
1. (Constant)	4.459	0.431		10.35	.000**
Age	–0.001	0.010	–0.004	–0.08	0.936
Victimization total score	0.011	0.003	0.229	4.20	.000**
Black (dummy variable)	–0.741	0.260	–0.167	–2.85	0.005**
Latina (dummy variable)	–0.515	0.197	–0.15	–2.62	0.009**
White (dummy variable; reference)	0.580	0.182	0.173	3.19	.002**
2. (Constant)	4.005	0.406		9.877	.000**
Age	.001	.010	.006	.110	.913
Victimization total score	0.011	.003	.213	3.866	.000**
CR	0.577	.214	0.160	2.692	.007**
Black dummy	–.647	.448	–0.146	–1.445	.149
Black dummy × CR	.226	.530	.044	0.427	.670
3. (Constant)	4.21	.430		9.794	.000**
Age	–.003	.010	–.014	–0.258	.797
Victimization total score	.011	.003	.229	4.183	.000**
CR	.285	.246	.079	1.158	.248
Latina dummy	.210	.350	.062	.599	.549
Latina dummy × CR	.627	.412	–0.176	1.521	.129
3. (Constant)	3.808	.402		9.479	.000**
Age	–.003	.010	–0.015	–.285	0.776
Victimization total score	.011	.003	.216	3.962	.000**
CR	.719	.272	.199	2.647	.009**
White dummy	.526	.390	.140	1.350	.178
White dummy × CR	.163	.323	.049	.505	.614

*Note.* 1 = housing instability by race; 2 = housing instability by CR; 3 = whether having a CR moderates the relationship between race and housing instability. **p < .01; *p < .05.

For Black DV survivors, CR did not moderate the relation between race and housing instability (B = .226, p = .670), though CR remained a significant predictor of housing instability (B = .577, p = .007). Similarly, for White DV survivors, CR did not moderate the relationship between race and housing instability (B = .526, p = .178), but having a CR remained a predictor of greater housing instability (B = .719, p = .009). Last, for Latina survivors, CR did not moderate the relationship between race and housing instability (B = –.627, p = .129) and CR was also not predictive of housing instability (Table 1).

## Discussion

This study is the first to demonstrate that unstably housed DV survivors with CRs who have sought out support services experience greater housing instability than those without a CR. This finding aligns with studies from the general population that have found that having a CR is associated with experiences of housing instability (Evans, 2016; Evans et al., 2019) and with studies with DV survivors that show that criminal behaviors are predictive of housing instability (Beijer et al., 2018). Prior studies on the relationship between CR and housing instability have found that housing discrimination may be a contributing factor to this relationship (Evans et al., 2019). Although having a CR was associated with experiencing more housing instability, within the current study the pathway of discrimination can only be inferred.

The unexpected finding that White women reported greater housing instability than did Women of Color is counter to evidence from general population studies and requires additional research. The finding may be related to selection bias within our sample. Since recruitment for the study involved reaching out to women who had already sought out help from DV agencies, it is possible that the unstably housed Women of Color in our sample are qualitatively different from and not representative of unstably housed Women of Color in the U.S. Women of Color are less likely to seek outside support when they are victims of DV than are White women (Grossman & Lundy, 2007). Potential reasons for this include fear or skepticism of institutions and agencies, and prior negative help-seeking experiences (Waller et al., 2021).

Additionally, all of the participants in this study were already unhoused or unstably housed. Many studies examining race differences on housing stability have compared stably housed to unhoused individuals (Desmond, 2016; Olivet et al., 2018), and these comparisons may be more likely to show race differences in the expected direction.

Next, we only asked if survivors had a CR that would show on a background check. We were not able to differentiate CR type (i.e., summary citations vs. misdemeanors vs. felonies), or type of offense (e.g., drug related, weapons charge) and whether the person has been incarcerated. Such variations in criminal history contribute meaningfully to variations in access to housing (Evans, 2016; Evans & Porter, 2014; Leasure & Martin, 2017). Finally, this study was cross-sectional, precluding the ability to examine causation.

This study was exploratory and preliminary. Despite its methodological limitations, it is the first to examine the critical associations among race, housing instability, and criminal record within a diverse, high-risk sample. Studies with larger sample sizes that include DV survivors who have never sought out DV support services and who have diverse socioeconomic and housing instability backgrounds, should be conducted to fully capture how these mechanisms function, so that appropriate interventions and policy changes can be implemented. Future work should also assess the role of variations in criminal record type in the relation between race and housing instability. Given the high rates of housing instability for this population, it is critical to identify and rectify individual factors impeding housing stability, so that survivors can better achieve safe and stable housing for themselves and their children.

## Author Biographies

**Jasmine Engleton**, MA, is a doctoral student at Michigan State University. Her research focuses on domestic violence survivors’ experience with housing instability as well as sexual assault survivor’s experience with CJ system. She continues to be interested in community-level responses to gender-based violence and how institutions and systems contribute to oppressive conditions (e.g., housing instability, homelessness, etc.).

**Noora Hamdan,** PhD, is a postdoctoral researcher at Michigan State University. She investigates how we can promote resilience in youth facing environmental and relational adversities in their everyday lives in the form of problematic parenting, housing instability, and domestic violence. She considers how fundamental adaptive systems can be leveraged to promote resilience in vulnerable individuals and groups.

**Cris M. Sullivan**, PhD, is Professor of Ecological/Community Psychology and Director of the Research Consortium on Gender-based Violence at Michigan State University. She has been an advocate and researcher in the movement to end gender-based violence since 1982. Her areas of expertise include developing and rigorously evaluating community interventions for abuse survivors and their children, and evaluating victim services.
